# Risk prediction of recurrent venous thromboembolism: a multiple genetic risk model

**DOI:** 10.1007/s11239-018-1762-7

**Published:** 2018-10-27

**Authors:** Abrar Ahmad, Kristina Sundquist, Karolina Palmér, Peter J. Svensson, Jan Sundquist, Ashfaque A. Memon

**Affiliations:** 1 Center for Primary Health Care Research, Department of Clinical Sciences, Lund University, Skåne University Hospital, Malmö, Sweden; 2 Department of Coagulation Disorders, Skåne University Hospital, Lund University, Malmö, Sweden; 3 Department of Family Medicine and Community Health, Department of Population Health Science and Policy, Icahn School of Medicine at Mount Sinai, New York, USA; 4 Center for Community-Based Healthcare Research and Education (CoHRE), Department of Functional Pathology, School of Medicine, Shimane University, Matsue, Japan; 5 Wallenberg Laboratory, 6th Floor, Inga Marie Nilssons Gata 53, 20502 Malmö, Sweden

**Keywords:** Cox regression analyses, Multiple SNPs model, Genetic variants, Recurrent VTE

## Abstract

A single genetic biomarker is unable to accurately predict the risk for venous thromboembolism (VTE) recurrence. We aimed to: (a) develop a multiple single nucleotide polymorphisms (SNPs) model to predict the risk of VTE recurrence and (b) validate a previously described genetic risk score (GRS) and compare its performance with the model developed in this study. Twenty-two SNPs, including established and putative SNPs associated with VTE risk, were genotyped in the Malmö thrombophilia study cohort (MATS; n = 1465, follow-up ~ 10 years) by using TaqMan PCR. Out of 22-SNPs, 12 had an association with the risk of VTE recurrence and were included for calculating GRSs. The risk of VTE recurrence was calculated by stratifying patients according to number of risk alleles. In 12-SNP GRS, patients with ≥ 7 risk alleles were associated with higher risk of VTE recurrence compared to patients having ≤ 6 risk alleles. In a simplified model (8-SNP GRS), the discriminative power of 8-SNP GRS was similar to that of 12-SNP GRS based on post-test probabilities (PP). Furthermore, 8-SNP GRS further improved the risk prediction of VTE recurrence in unprovoked VTE and male patients (PP% = 15.4 vs 8.3, 17.1 vs 7.2 and 19.0 vs 7.1 for high risk groups vs low risk groups in whole population, males and unprovoked VTE patients respectively). In addition, we also validated previously described 5-SNP GRS in our cohort and found that the 8-SNP GRS performed better than the 5-SNP GRS in terms of higher PP. Our results show that a multiple SNP GRS consisting of 8-SNPs may be an effective model for prediction of VTE recurrence, particularly in unprovoked VTE and male patients.

**Electronic supplementary material:**

The online version of this article (10.1007/s11239-018-1762-7) contains supplementary material, which is available to authorized users.

## Highlights

- A single genetic biomarker is unable to accurately predict the risk for VTE recurrence
- We genotyped important genetic variants in multiple genes associated with VTE in a prospective follow-up study of 1465 objectively diagnosed VTE patients.
- Our results showed that a genetic risk score (GRS) consisting of 8-single nucleotide polymorphisms (SNPs) can be an effective model for the prediction of VTE recurrence.
- We also validated previously described 5-SNP GRS in Swedish population and showed that 8-SNP GRS developed in this study had modestly improved discriminating performance than previously described 5-SNP model based on the post-test probabilities of high and low risk groups.

## Introduction

Venous thromboembolism (VTE) that comprises deep vein thrombosis (DVT) and pulmonary embolism (PE) is associated with significant rate of morbidity, mortality, substantial health-care costs and high rate of recurrence. In Europe, an estimated annual incidence rate of VTE ranges from 1 to 2 per 1000 person-years that is comparable to the incidence rate of stroke [1–3]. Patients diagnosed with primary VTE are always at the risk for recurrence, irrespective of the time elapsed since the primary event, nevertheless, the risk of recurrence does decline over time. About 20–30% of primary VTE patients develop recurrence within 5-years after diagnosis [4, 5].

The risk of recurrence in unprovoked primary VTE patients is 2–3 fold higher as compared to provoked VTE i.e., VTE with transient risk factors (surgical intervention, immobilization or cast therapy within the last month, female hormone therapy, use of contraceptives pills, current pregnancy and postpartum period [first 6 weeks after delivery]) and malignancies [5–8]. Taking into consideration the high rates of morbidity and mortality, prediction and prevention of VTE recurrence is indispensable. Continued anticoagulation after the primary VTE decreases the risk of recurrence, however, it should be carefully weighed against the risk of serious side effects i.e. anticoagulant-related bleeding [9–11].

VTE is a multifactorial and complex disease influenced by several genetic and non-genetic risk factors. If the cause of primary VTE is genetic, the risk of recurrence is lifelong. Familial studies have estimated the heritability of VTE at about 50–60% [12, 13]. Previously known genetic risk factors i.e. heritable thrombophilia: *factor V* Leiden, *factor II* G20210A mutation, protein C, protein S and antithrombin deficiency, does not appear to predict the risk for recurrent VTE [14]. Likewise, Bezemer et al., and Sundquist et al., reported that despite the presence of known genetic risk factors, family history is still a significant risk factor for primary and recurrent VTE, suggesting the presence of additional unidentified genetic risk factors [15, 16].

Unfortunately, no single genetic biomarker can accurately predict the risk of VTE recurrence, therefore, it necessitates the risk classification based on multiple genetic biomarkers to precisely predict the risk of VTE recurrence [17, 18]. For the risk prediction of VTE recurrence, Van Hylckama Vlieg et al. analyzed 31-SNPs from 22 genes that have been previously investigated for their role in risk assessment of primary VTE [19]. They proposed a simplified genetic risk score model (5-SNP GRS) i.e. rs6025, rs1799963, rs8176719, rs2066865, and rs2036914, which could adequately stratify patients with high and low risk of VTE recurrence. However, this model remains to be validated in an independent cohort.

To examine as to what extent the VTE related genetic variants can be used to predict the risk of VTE recurrence in whole population as well as in high-risk groups, i.e., unprovoked and in male VTE patients, we investigated 22-SNPs in 16 VTE associated genes. These genes are associated with various biological pathways (lipid metabolism, thrombosis and hemostasis, inflammation etc.) implicated in the pathogenesis and/or pathophysiology of VTE [20–25]. In this study, we analyzed these 22 genetic variants in the MATS cohort to: (a) develop a multiple SNPs model to predict the risk of VTE recurrence and (b) validate a previously described genetic risk model and compare its performance with a model developed in this study.

## Materials and methods

### Study population

Malmö thrombophilia study (MATS; n = 1465) is a population based, prospective follow up study in which objectively diagnosed VTE patients at Skåne University Hospital Malmö, were included. MATS patients were followed from the time of inclusion until diagnosis for VTE recurrence, death or end of the study (1998–2008). The study design and complete information about MATS (including treatment, inclusion, exclusion criteria etc.) has been described elsewhere [26, 27]. In short, inclusion criteria for MATS were: age > 18 years, objective diagnosis for VTE and/or PE with one or more of the following methods i.e. phlebography, computed tomography (CT), lung scintigraphy, duplex ultrasonography and magnetic resonance imaging (MRI) and willing to participate in MATS. The rate of consensual participation in MATS was 70% and remaining 30% patients did not participate due to one or more of the following reasons: language problems, presence of other severe diseases, dementia and/or unwillingness to participate in MATS. Thrombophilia was defined as the presence of the factor V Leiden (FVL, rs6025) or *factor II* G20210A mutation (rs1799963) or a level below the laboratory reference range of protein C (< 0.7 kilo international unit (kIU)/L) or free protein S (female < 0.5 kIU/L, male < 0.65 kIU/L) or antithrombin (< 0.82 kIU/L) in VTE patients without anticoagulant treatment. MATS patients were treated according to the standard treatment protocol at Malmö University Hospital, i.e., low-molecular weight heparin (LMWH) or unfractionated heparin (UFH) during the initiation of oral anticoagulants (until international normalized ratio [INR] value is ≥ 2.0 but at least 5 days). According to Malmö University Hospital treatment protocol recommendations, VTE patients will get 3–6 months of oral anticoagulant therapy for first-time VTE with the consideration of extension of treatment if VTE recurrence occurs. Family history of VTE was defined as a history of VTE in first-degree relatives (son/daughter, sibling, or parent). Data on recurrent VTE and mortality was obtained from the patient files. Unprovoked VTE was defined as VTE in patients without acquired risk factors for VTE (surgical intervention, immobilization or cast therapy within the last month, female hormone therapy, use of contraceptives pills, current pregnancy and postpartum period [first 6 weeks after delivery]) and malignancies diagnosed prior to or at diagnosis of the first VTE event.

The follow-up period in MATS (mean ± SD, 3.9 ± 2.5 years) was calculated after stopping the anticoagulant treatment and until the diagnosis for VTE recurrence, death of the patient or end of the study (December 2008).

### Ethical permission

All the patients provided a written consent according to the declaration of Helsinki. The study was approved by the Medical Ethical Committee of the Lund University.

### DNA extraction and genotype determination

From the whole blood, genomic DNA was extracted by using QiAmp 96 DNA Blood Kit (Qiagen, Hilden, Germany) according to the supplier’s instructions. Pre-designed or, in some cases, custom made TaqMan® Genotyping Assays were designed and used for genotyping (with VIC and FAM probes). Genotyping of selected SNPs was performed by TaqMan PCR according to the manufacturer’s protocol (Applied Biosystems, Life Technologies Corporation, Carlsbad, CA, USA). To determine various alleles in investigated polymorphisms, BioRad CFX manager software was used. A full list of SNPs included in this study is presented in Table 2.

A chromogenic method using Berichrom® Protein C reagent (Siemens Healthcare Diagnostics, Upplands Väsby, Sweden) was used to analyse the Protein C activities [28]. Analysis of free Protein S antigen concentration was performed by latex immunoassay with Coamatic® Protein S-Free (Chromogenix, Haemochrom Diagnostica AB, Gothenburg, Sweden) [29]. Antithrombin III antigen concentration was analyzed by a thrombin-based method using Berichrom Antithrombin reagent (Siemens Healthcare Diagnostics) [30].

### Statistical approach

Chi square test was used to compare dichotomous variables while Student t-test (if data was normally distributed) or Mann–Whitney *U* test (if data was not normally distributed) to compare continuous variables. For normally distributed variable, data was presented as mean ± SD whereas for the skewed variable, data was presented as median and IQR (interquartile range). To examine individual associations of all 22-SNPs with the risk of recurrent VTE, univariate and multivariate (adjusted for age, sex and family history of VTE) Cox regression analyses were performed (Table 2). Genotypes in all SNPs were analyzed and presented either as recessive [homozygous wild type (0) plus heterozygous (1) and compared with homozygous mutated form (2)] or dominant models (homozygous wild-type compared with heterozygous plus homozygous mutated form).

**Fig. 1:**
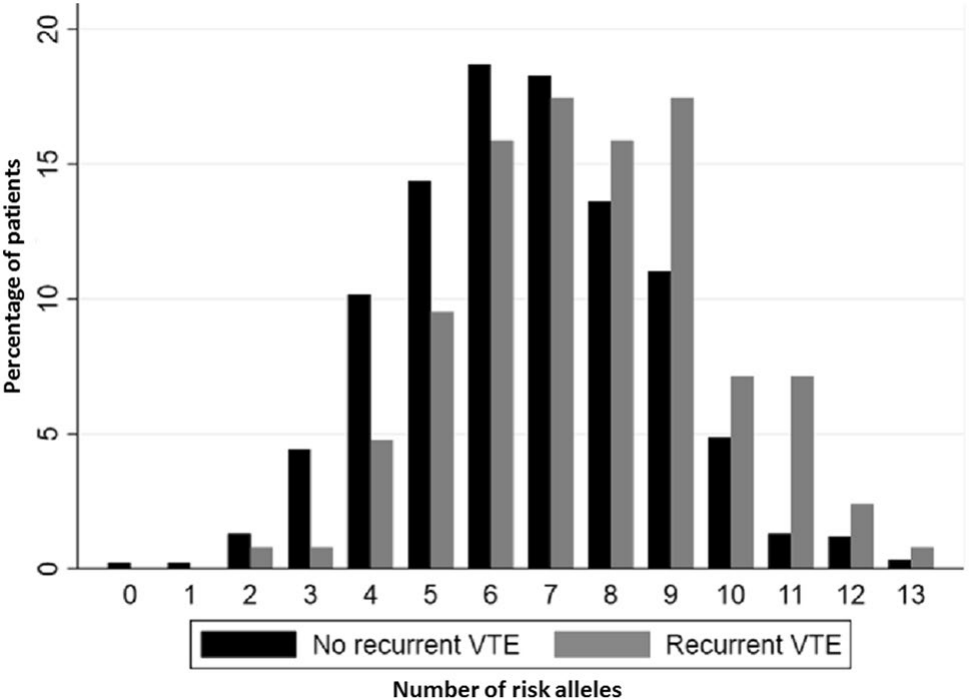
The 12-single nucleotide polymorphisms and their risk alleles distribution among recurrent and non-recurrent VTE patients

The number of risk alleles of the individual SNPs with a positive association to recurrent VTE (HR > 1.2) were summed to generate a genetic risk score (10 out of the 22-SNPs were excluded because of too low HR). Three different genetic risk scores (GRSs) were developed by summing different number of SNPs; 12-SNPs; 8-SNPs and 5-SNPs, a previously described model [19]. The distribution of the number of risk alleles for 12-SNP GRS is shown in Fig. 1 separated by recurrent and non-recurrent VTE.

The three GRSs were dichotomized into two groups (low and high risk) to evaluate their predictive ability, as measured by HRs (high vs low) and post-test probabilities (for low risk group as well as for high risk group). HRs were estimated to yield a measure of the association to the risk of recurrent VTE and post-test probabilities (predictive values after fitted logistic regression models) to estimate the possibility to differentiate recurrent from non-recurrent VTE. These probabilities were compared to a pre-test probability, calculated as the prevalence of recurrent VTE (number of individuals with recurrent VTE divided by total number of individuals). The pre- and post-test probabilities are presented which show the percentages of patients at risk of VTE recurrence before and after the application of genetic risk score respectively.

Confidence intervals of post-test probabilities were calculated by normal approximation with standard errors estimated using the delta method. The three models were evaluated in whole population, for males and females, and for provoked and unprovoked VTE patients (Table 3).

**Fig. 2:**
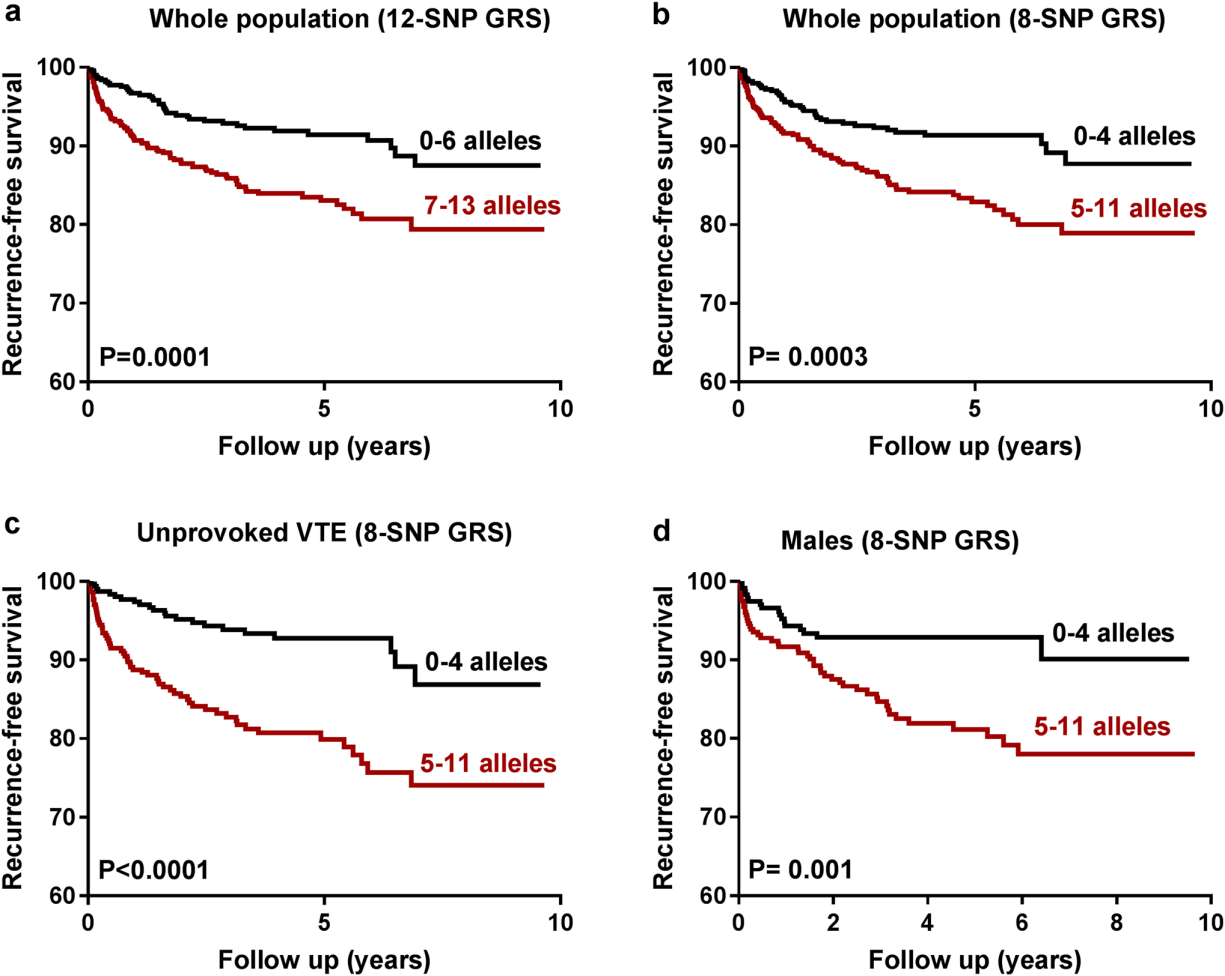
**a** Survival curves showing recurrence-free survival for the 12-SNP GRS stratified in high and low risk of VTE recurrence in the whole population, **b** Survival curves showing recurrence-free survival for the 8-SNP GRS stratified in high and low risk of VTE recurrence in the whole population, **c** Survival curves showing recurrence-free survival for the 8-SNP GRS stratified in high and low risk of VTE recurrence in unprovoked VTE and, **d** Survival curves showing recurrence-free survival for the 8-SNP GRS stratified in high and low risk of VTE recurrence in male patients

Kaplan–Meier curves were plotted for 12-SNPs and 8-SNP GRSs to calculate recurrence-free survival in whole population and for males and unprovoked VTE patients (Fig. 2). To assess the goodness-of-fit of these models, Akaike information criteria (AIC) and Harrell’s Concordance Index (Harrell’s C) were calculated. STATA version 15 (StataCorp LP) and SPSS version 21 (IBM, Armonk, NY, USA) were used for all statistical analyses.

## Results

Of the 1465 patients in this cohort, the patients who had one or more thrombotic events before the start of this study (n = 154) were excluded because the follow-up period was started after stopping the anticoagulant treatment and we do not have information about the treatment and follow-up period of pervious episodes of VTE in these patients. Among the remaining patients (n = 1311), 148 had VTE recurrence during the follow-up period. By comparing recurrent and non-recurrent VTE patients, we found that the frequency of thrombophilia was significantly higher (49.6%) in recurrent VTE patients as compared to those with non-recurrent VTE (36.9%). Moreover, 32.4% of recurrent VTE patients had a family history of VTE as compared to 23.5% in non-recurrent VTE patients. Higher number of male patients had VTE recurrence compared to the female patients, though the difference didn’t reach statistical significance. However, stratification according to age showed that the number of male patients ≤ 45 years of age was significantly higher in the recurrent group compared to female patients with ≤ 45 years of age. However, no difference was found between male and female patients above > 45 years of age. No significant differences were found between recurrent and non-recurrent VTE patients in the other variables, i.e., BMI, DVT, PE, malignancy and other acquired risk factors for VTE (Table 1).

**Table 1:** Basic characteristics of the study population stratified by recurrent and non-recurrent VTE patients

Parameters	Non recurrent VTE n (%)	Recurrent VTE n (%)	Total n (%)	P-value^c^
Age at inclusion				
Years, median (IQR)	66.4 (24)	63.4 (24)	65.8 (24)	0.088^b^
BMI				
Mean ± SD	26.6 ± 4.7	27.4 ± 5.1	26.6 ± 4.8	0.066^a^
Sex (age ≤ 45 years)				
Males	67 (33.7)	16 (61.5)	83 (36.9)	0.009
Females	132 (66.3)	10 (38.5)	142 (63.1)	
(Age > 45 years)				
Male	498 (51.7)	62 (50.8)	560 (51.6)	0.923
Female	466 (48.3)	60 (49.2)	526 (48.4)	
DVT				
DVT	886 (76.2)	116 (78.4)	1002 (76.4)	0.608
No DVT	277 (23.8)	32 (21.6)	309 (23.6)	
PE				
PE	343 (29.5)	45 (30.4)	388 (29.6)	0.848
No PE	820 (70.5)	103 (69.6)	923 (70.4)	
DVT + PE				
DVT	820(70.5)	103 (69.6)	923 (70.4)	0.306
PE	277 (23.5)	32 (21.6)	309 (23.6)	
DVT + PE	66 (5.7)	13 (8.8)	79 (6)	
Malignancy				
Yes	140 (12.1)	13 (8.8)	153 (11.7)	0.278
No	1020 (87.9)	135 (91.2)	1155 (88.3)	
Thrombophilia				
Yes	390 (36.9)	68 (49.6)	458 (38.3)	0.005
No	668 (63.1)	69 (50.4)	737 (61.7)	
Acquired risk factors				
Yes	499 (42.9)	53 (35.8)	552 (42.1)	0.112
No	664 (57.1)	95 (64.2)	759 (57.9)	
Family history				
Yes	269 (23.5)	47 (32.4)	316 (24.5)	0.024
No	875 (76.5)	98 (67.6)	973 (75.5)	

*DVT* deep vein thrombosis, *PE* pulmonary embolism, *BMI* body mass index, *IQR* interquartile range

^a^Student T-test

^b^Mann-Whitney U test

^c^Comparing non-recurrent with recurrent VTE

For the risk assessment analyses of recurrent VTE among the 1311 VTE patients, those patients who had VTE recurrence during anticoagulant treatment, those who died during anticoagulant treatment or those for whom complete information was missing were excluded (n = 260). Of the remaining 1051 patients, 126 (12%) had recurrent VTE and 925 were recurrence-free during the follow-up period and these were included for the risk assessment analyses.

### Risk of VTE recurrence in 12-SNP GRS

The individual association of each of 22-SNPs with the risk of VTE recurrence is shown in Table 2. Among these SNPs, the strongest positive associations were observed for *A2M* (rs3832852), *ABO* (rs8176719), *FGG* (rs2066865) and FVL (HR 2.60, 1.97, 1.77 and 1.62 respectively, adjusted for sex, age and family history of VTE). All other SNPs showed mild, no, or negative association with the risk of VTE recurrence when analyzed individually. Furthermore, to investigate whether increasing the number of risk alleles can increase the risk of VTE recurrence, we first developed a genetic risk score based on 12-SNPs that had a significant or trend of a positive association with the risk of VTE recurrence (Table 2). The total number of risk alleles in all VTE patients analyzed in the study ranged from 0 to 13. A trend of increasing number of patients at risk of recurrence was observed with increasing number of risk alleles (Fig. 1). Thus, by using a 12-SNP GRS, we were able to stratify patients in low and high risk groups. Patients having ≥ 7 risk alleles were at higher risk of VTE recurrence compared to those with ≤ 6 risk alleles (HR 2.06, 95% CI 1.41–2.99). We also tested the discriminatory performance of 12-SNP GRS in terms of post-test probabilities and showed that the high risk group stratified by 12-SNP GRS had a higher probability of VTE recurrence than the low risk group (PP% for high risk group = 15.5, 95% CI 12.5–18.5 vs PP% for low risk group = 8.0, 95% CI 5.7–10.4) (Table 3).

**Table 2:** Cox regression analyses for all individual SNPs using the number of genotypes dichotomized (uni- and multi-variate analyses)

Gene	Pathways (established or potential, leading to VTE)	SNP	Dichotomization of genotypes	Univariate	Adjusted for sex, age and family history of VTE
				HR (95% CI)	HR (95% CI)
*A2M*	Coagulation	rs3832852	2 vs 0 + 1	2.66 (1.08–6.52)	2.60 (1.06–6.40)
*ABO*	Coagulation	rs8176719	1 + 2 vs 0	2.05 (1.21–3.46)	1.97 (1.16–3.34)
*FGG*	Coagulation	rs2066865	2 vs 0 + 1	1.82 (1.11–3.01)	1.77 (1.07–2.93)
*FV*	Coagulation	rs6025	1 + 2 vs 0	1.71 (1.20–2.44)	1.62 (1.12–2.33)
*PAI-1*	Fibrinolytic	rs1799889	1 + 2 vs 0	1.59 (1.06–2.38)	1.58 (1.05–2.38)
*F11*	Coagulation	rs2036914	2 vs 0 + 1	1.51 (1.00-2.29)	1.54 (1.02–2.34)
*FTO*	Lipid metabolism	rs9939609	2 vs 0 + 1	1.40 (0.93–2.12)	1.45 (0.96–2.20)
*FII*	Coagulation	rs1799963	1 + 2 vs 0	1.39 (0.68–2.85)	1.39 (0.67–2.85)
*ApoM*	Lipid metabolism	rs805297	1 + 2 vs 0	1.34 (0.94–1.92)	1.36 (0.95–1.94)
*MRPL37*	mtDNA regulation	rs10888838	1 + 2 vs 0	1.32 (0.89–1.96)	1.29 (0.86–1.93)
*THBD*	Coagulation	rs1042580	1 + 2 vs 0	1.29 (0.90–1.86)	1.26 (0.87–1.82)
*TFAM*	mtDNA regulation	rs1937	1 + 2 vs 0	1.25 (0.84–1.85)	1.24 (0.83–1.84)
*THBD*	Coagulation	rs3176123	1 + 2 vs 0	1.12 (0.79–1.60)	1.11 (0.78–1.59)
*THBD*	Coagulation	rs1042579	1 + 2 vs 0	1.12 (0.78–1.59)	1.11 (0.77–1.58)
*P-Selectin*	Inflammation	rs6136	1 + 2 vs 0	1.01 (0.66–1.55)	1.04 (0.67–1.60)
*IL1RL1*	Inflammation	rs3821204	1 + 2 vs 0	0.93 (0.65–1.32)	0.94 (0.66–1.34)
*THBD*	Coagulation	rs1962	1 + 2 vs 0	0.90 (0.63–1.29)	0.92 (0.64–1.31)
*TLR9*	Inflammation	rs5743836	1 + 2 vs 0	0.87 (0.58–1.31)	0.85 (0.56–1.28)
*PARK2*	mtDNA regulation	rs4708928	1 + 2 vs 0	0.84 (0.58–1.23)	0.80 (0.54–1.16)
*ApoM*	Lipid metabolism	rs9404941	1 + 2 vs 0	0.80 (0.46–1.39)	0.81 (0.46–1.41)
*IL1RL1*	Inflammation	rs950880	1 + 2 vs 0	0.78 (0.55–1.10)	0.78 (0.55–1.11)
*FII*	Coagulation	rs3136520	1 + 2 vs 0	0.74 (0.33–1.68)	0.77 (0.34–1.75)

*HR* hazard ratio of recurrent VTE for high vs low, 0 = wildtype homozygous, 1 = heterozygous, 2 = mutant homozygous, mtDNA = mitochondrial DNA

**Table 3:** Predictive ability of different dichotomized models

Model	Low risk group (n, %)	High risk group (n, %)	HR (95% CI)	Pre-test probability (%)	Post-test probability (%) low risk group	Post-test probability (%) high risk group
Whole population						
12-SNP GRS	0–6 (n = 497, 47.3%)	7–13 (n = 554, 52.7%)	2.06 (1.41–2.99)	12.0	8.0 (5.7–10.4)	15.5 (12.5–18.5)
8-SNP GRS	0–4 (n = 507, 48.2%)	5–11 (n = 544, 51.8%)	1.94 (1.34–2.81)	12.0	8.3 (5.9–10.7)	15.4 (12.4–18.5)
5-SNP GRS	0–2 (n = 456, 43.4%)	3–7 (n = 595, 56.6%)	1.61 (1.11–2.34)	12.0	9.2 (6.6–11.9)	14.1 (11.3–16.9)
Males						
12-SNP GRS	0–6 (n = 235, 45.5%)	7–13 (n = 282, 54.5%)	2.42 (1.39–4.21)	12.6	7.2 (3.9–10.5)	17.0 (12.6–21.4)
8-SNP GRS	0–4 (n = 236, 45.6%)	5–11 (n = 281, 54.4%)	2.42 (1.39–4.21)	12.6	7.2 (3.9–10.5)	17.1 (12.7–21.5)
5-SNP GRS	0–2 (n = 208, 40.2%)	3–6 (n = 309, 59.8%)	1.60 (0.94–2.71)	12.6	9.6 (5.6–13.6)	14.6 (10.6–18.5)
Females						
12-SNP GRS	0–6 (n = 262, 49.1%)	7–13 (n = 272, 50.9%)	1.78 (1.06–2.98)	11.4	8.8 (5.4–12.2)	14.0 (9.9–18.1)
8-SNP GRS	0–4 (n = 271, 50.7%)	5–10 (n = 263, 49.3%)	1.59 (0.95–2.64)	11.4	9.2 (5.8–12.7)	13.7 (9.5–17.8)
5-SNP GRS	0–2 (n = 248, 46.4%)	5–11 (n = 286, 53.6%)	1.62 (0.96–2.73)	11.4	8.9 (5.3–12.4)	13.6 (9.7–17.6)
Unprovoked VTE						
12-SNP GRS	0–6 (n = 303, 48.9%)	7–13 (n = 316, 51.1%)	2.29 (1.43–3.68)	13.1	8.3 (5.2–11.3)	17.7 (13.5–21.9)
8-SNP GRS	0–4 (n = 309, 49.9%)	5–11 (n = 310, 50.1%)	2.83 (1.73–4.61)	13.1	7.1 (4.3–10.0)	19.0 (14.7–23.4)
5-SNP GRS	0–2 (n = 279, 45.1%)	3–7 (n = 340, 54.9%)	2.24 (1.38–3.63)	13.1	8.2 (5.0-11.5)	17.1 (13.1–21.1)
Provoked VTE						
12-SNP GRS	0–6 (n = 194, 44.9%)	7–13 (n = 238, 55.1%)	1.70 (0.92–3.17)	13.1	7.7 (4.0-11.5)	12.6 (8.4–16.8)
8-SNP GRS	0–4 (n = 198, 45.8%)	5–11 (n = 234, 54.2%)	1.07 (0.59–1.92)	13.1	10.1 (5.9–14.3)	10.7 (6.7–14.6)
5-SNP GRS	0–2 (n = 177, 41.0%)	3–7 (n = 255, 59.0%)	0.96 (0.53–1.73)	13.1	10.7 (6.2–15.3)	10.2 (6.5–13.9)

Models were calculated by summing the number of risk alleles for different SNPs. The sum is dichotomized into ‘low’ and ‘high’ risk groups (Univariate analyses)

12-SNP GRS: [*FII* (rs1799963), *FV* (rs6025), *ABO* (rs8176719), *ApoM* (rs805297), *F11* (rs2036914), *FGG* (rs2066865), *MRPL37* (rs10888838), *THBD* (rs1042580), *A2M* (rs9939609), *FTO* (rs9939609*), PAI-1* (*rs1799889*), *TFAM* (*rs1937*)]]. 8-SNP GRS: [*FII* (rs1799963), *FV* (rs6025), *ABO* (rs8176719), *ApoM* (rs805297), *F11* (rs2036914), *FGG* (rs2066865), *PAI-1* (*rs1799889*), *TFAM* (*rs1937*)]

5-SNP GRS, previously described by Van Hylckama et al. (2014): [*FII* (rs1799963), *FV* (rs6025), *ABO* (rs8176719), *F11* (rs2036914), *FGG* (rs2066865)]

*HR* hazard ratio of recurrent VTE, *Pre-test probability* risk of recurrent VTE before SNPs model, *Post-test probability* risk of recurrent VTE after SNPs model, *n* number of risk alleles, *Unprovoked VTE* VTE patients with no acquired risk factors for VTE and no malignancy

We also adjusted the 12-SNP GRS with sex, age and family history of VTE and the results remained unchanged (Supplementary Table 1).

We further stratified patients according to low, medium and high number of risk alleles and the results showed that the risk of VTE recurrence increased with increasing numbers of risk alleles. The probability of VTE recurrence was highest in patients having ≥ 10 risk alleles (PP%=23.7, 95% CI 15.0–32.3) compared to patients having 6–9 (PP% = 12.8, 95% CI 10.3–15.4) and 0–5 risk alleles (PP% = 6.6, 95% CI 3.8–9.4) (Supplementary Table 2).

### Risk of VTE recurrence in 8-SNP GRS

To develop a genetic risk score using a more parsimonious model, we tested various combinations by adding SNPs one by one. Our results showed that 8-SNP GRS [*FII* (rs1799963), *FV* (rs6025), *ABO* (rs8176719), *ApoM* (rs805297), *F11* (rs2036914), *FGG* (rs2066865), *PAI-1* (rs1799889) and *TFAM* (rs1937)] had similar discrimination power as that of the 12-SNP GRS in terms of post-test probabilities (PP% for high risk group = 15.5 vs 15.4 for 12-SNP vs 8-SNP GRSs). The number of risk alleles in 8-SNP GRS ranged from 0 to 11 and the patients were stratified as high and low risk groups according to the presence of total risk alleles. Patients having ≥ 5 risk alleles had a higher risk of VTE recurrence as compared to patients with ≤ 4 risk alleles (PP% for high risk group = 15.4, 95% CI 12.4–18.5 vs PP% for low risk group = 8.3, 95% CI 5.9–10.7). Patients having ≥ 5 risk alleles were at higher risk of VTE recurrence compared to those with ≤ 4 risk alleles (HR 1.94, 95% CI 1.34–2.81) (Table 3).

We further stratified patients according to a low, medium and high number of risk alleles and the results showed that the risk of VTE recurrence increased with increasing numbers of risk alleles. The probability of VTE recurrence was highest in patients having ≥ 8 risk alleles (PP% = 28.3, 95% CI 15.2–41.3) compared to patients having 4–7 (PP% = 13.2, 95% CI 10.8–15.7) and 0–3 risk alleles (PP% = 6.3, 95% CI 3.5–9.1) (Supplementary Table 2).

### Risk prediction of VTE recurrence according to sex

In sub-analyses, we stratified VTE patients according to sex to investigate the performance of 8-SNP GRS for risk assessment of VTE recurrence. Male patients having ≥ 5 risk alleles had significantly higher risk (HR 2.42, 95% CI 1.39–4.21) of VTE recurrence than those having ≤ 4 risk alleles (Reference) (Table 3). Furthermore, the discriminating performance of 8-SNP GRS was improved in male patients (PP% for high risk group = 17.1, 95% CI 12.7–21.5) compared to the whole population (PP% for high risk group = 15.4, 95% CI 12.4–18.5) while in female patients no improvement in discriminatory power of the 8-SNP GRS was observed based on post-test probability (PP% for high risk group = 13.7, 95% CI 9.5–17.8) (Table 3). Further stratification of patients into three categories i.e. low, medium and high was not possible due to the low number of patients in this subgroup.

### Risk prediction of VTE recurrence in unprovoked VTE patients

In the sub-analyses of unprovoked first VTE patients (n = 619), the risk of recurrence was higher in patients having ≥ 5 risk alleles (HR 2.83, 95% CI 1.73–4.61) compared to those having ≤ 4 risk alleles (reference). The discriminating performance of the 8-SNP GRS was further improved in unprovoked VTE patients in terms of post-test probabilities (PP% = 19.0, 95% CI 14.7–23.4 vs PP% = 7.1, 95% CI 4.3–10.0 for high vs low risk groups, respectively).

In provoked VTE patients however, the discriminating performance of the 8-SNP GRS was not improved in the high risk group as compared to the low risk group in terms of post-test probabilities (PP% = 10.7, 95% CI 6.7–14.6 vs PP% = 10.1, 95% CI 5.9–14.3) Table 3. Further stratification of patients into three categories i.e. low, medium and high was not possible due to the low number of patients in this subgroup.

### Validation of the previously described genetic risk score and comparison with the 8-SNP GRS

We also validated the previously described 5-SNP GRS, (rs6025, rs1799963, rs8176719, rs2036914 and rs2066865), for the risk assessment of VTE recurrence by van Hylckama Vlieg et al. [19]. We found that the previously described 5-SNP GRS was also efficient in discrimination of patients in the whole population (PP% for high risk group patients = 14.1, 95% CI 11.3–16.9) as well as in unprovoked VTE (PP% for high risk group patients = 17.1, 95% CI 13.1–21.1) and male VTE patients (PP% for high risk group patients = 14.6, 95% CI 10.6–18.5) (Table 3).

Comparison between the previously described 5-SNP GRS and the 8-SNP GRS presented in this study showed that the discriminatory performance of the 8-SNP GRS was modestly improved in terms of higher post-test probabilities in the whole population (8-SNP GRS vs 5-SNP GRS, PP%; 15.4 vs 14.1) as well as in unprovoked VTE (8-SNP GRS vs 5-SNP GRS, PP%; 19.0 vs 17.1) and male patients (8-SNP GRS vs 5-SNP GRS, PP%; 17.1 vs 14.6) (Table 3).

To measure how well the risk score predicts the distribution of time in the Cox regression models as well as to estimate the quality of the model relative to the others, Harrell’s C and AIC were calculated, respectively (data not shown).

Kaplan–Meier curves were plotted to calculate the recurrence-free survival for the 12-SNPs and 8-SNP GRS. Patients having ≥ 7 risk alleles had a shorter recurrence-free survival as compared to patients having ≤ 6 risk alleles in the 12-SNP GRS (Fig. 2a). Similar results were found in the 8-SNP GRS for the whole population as well as for unprovoked first VTE and male patients, where a high number of risk alleles was associated with a shorter recurrence-free survival than a low number of risk alleles (Fig. 2b–d respectively).

## Discussion

In the current study, we investigated the role of 22-SNPs from 16 genes for the risk assessment of recurrent VTE. We have developed a GRS based on 8-SNPs [*FII* (rs1799963), *FV* (rs6025), *ABO* (rs8176719), *ApoM* (rs805297), *F11* (rs2036914), *FGG* (rs2066865), *PAI-1* (rs1799889) and *TFAM* (rs1937)] and showed that this model is effective in discriminating VTE patients into high and low risk groups. The discriminatory power of the 8-SNP GRS was even stronger in male and unprovoked VTE patients. We also validated previously described 5-SNP GRS and compared it’s performance with our 8-SNP GRS and the results showed that our 8-SNP GRS had a modestly improved performance in discriminating patients with a higher risk of VTE recurrence in terms of higher post-test probabilities [19].

Efforts have been made to develop risk models based on genetic risk scores for better prediction of VTE [17–19, 31]. In agreement with our findings, some previous studies have also found that an increase in the number of risk alleles in the prediction model significantly improves the risk prediction of VTE recurrence [18, 19].

Genetic variants in the 8-SNP GRS represent genes that are associated with the pathophysiology of VTE. *Factor V* and *Factor II* are integral parts of the thrombotic pathway [32]. Having an *ABO* blood group was reported to be involved in thrombosis, affecting *factor VIII* and von *Willebrand factor* levels [33]. *FGG*, being the precursor of fibrin, is an essential component of the hemostatic system [34]. In previous studies, we have shown that genetic variants and protein levels of ApoM were associated with the risk of VTE recurrence in a sex-specific manner [22, 26]. Similarly, *PAI-1* polymorphism has been reported by us to be associated with an increased risk of VTE recurrence in the presence of FVL [35]. We have recently shown an association between risk of VTE recurrence and SNPs in mitochondrial regulating genes, suggesting a potential role of mitochondrial function in VTE [36]. Mitochondrial transcription factor A (*TFAM*) has been shown to be involved in mitochondrial biogenesis that in turn affects inflammation; a well-known mechanism in thrombosis [37–39]. The association between *TFAM* polymorphism and the risk of VTE recurrence warrants, however, further investigation of mitochondrial regulating genes in VTE.

The risk of VTE recurrence is higher after stopping anticoagulant therapy and proper selection of patients with the highest risk of VTE recurrence for long-term anticoagulant therapy is important. Although FVL and *FII* G20210A are the most important genetic variants associated with the risk of primary VTE, both genetic variants are unable to precisely predict the risk of recurrent VTE [14, 40]. We investigated 22-SNPs (including FVL and *FII* G20210A) related to VTE and present a GRS which can be a useful model for prediction of VTE recurrence.

VTE recurrence is higher in patients with unprovoked first VTE as compared to those with provoked, i.e., those having acquired risk factors for VTE [7, 41]. We investigated our 8-SNP GRS in provoked and unprovoked VTE and found that the discriminatory performance in identifying patients at higher risk of VTE recurrence was improved in terms of post-test probability in unprovoked VTE patients. However, in provoked VTE patients, 8-SNP GRS was unable to stratify patients into high and low risk of VTE recurrence. The possible explanation for these results could be that in provoked VTE patients, the cause of VTE is not genetic rather it is the provoking factor which increases the risk and once that risk factor is removed, patients are no longer at the risk of VTE recurrence. In agreement with our findings, previous studies also showed that the risk of VTE recurrence is lower in those VTE patients whom first episode was provoked by transient risk factors [42]. Furthermore, sex is an important risk factor of VTE recurrence and male patients have approximately 2–4 fold higher risk of VTE recurrence as compared to females [43–45], though the pathophysiology underlying this phenomenon is still unknown. Our results showed that higher number of risk alleles in male patients was associated with higher risk of VTE recurrence and had better discriminatory performance than in female patients. These findings are consistent with previous reports that male and female patients have different risk factors for VTE recurrence [46] and our findings may partly suggest an explanation for the higher risk of VTE recurrence in males.

We also validated a previously described genetic risk model in our patient cohort [19]. Furthermore, we showed that the 8-SNP GRS had a modestly better risk prediction performance in the whole population as well as those with unprovoked VTE and male patients, compared to the previously described 5-SNP GRS. Both GRSs (8-SNPs, and 5-SNPs) had five SNPs in common. The improved discriminative performance for the 8-SNP GRS may possibly be due to the inclusion of 3 additional SNPs; however, our findings need to be validated in other cohorts as well.

One of the limitation of the current study is that it does not include patients ≤ 18 years of age; therefore, our model cannot be used for patients ≤ 18 years. Secondly, the current study was performed in Malmö and surrounding areas and therefore its validity may be applied only in patients with similar ethnic background, i.e., Swedish. Nevertheless, in this study we have not only validated a previously described GRS but also improved its performance in terms of prediction value in our new model.

The common genetic variants are usually located in non-coding regions of the chromosome, which don’t code for specific functional proteins and therefore may have a weaker effect on VTE. By contrast, variants at exonic regions of the chromosome, which code for functional proteins are often rare, but may have a greater impact on disease development. The recent increase in studies based on exome and/or whole-genome sequencing will further identify novel genetic risk variants, which may, together with clinical risk factors, further improve the risk prediction of VTE recurrence [47, 48].

The 8-SNP GRS developed in this study can differentiate patients into low and high risk of VTE patients. If validated, this model might be integrated for consideration in designing individualized therapeutic strategies for VTE patients; low risk group may stop anticoagulant treatment after initial treatment period while high risk group may continue anticoagulant therapy for a longer period.

In conclusion, we showed that 8-SNP GRS can be an effective model for prediction of VTE recurrence. Its performance was further improved in patients with high risk of VTE recurrence i.e., males and unprovoked VTE patients. However, to confirm the rationality of the presented model, validation in other cohorts is needed.

## Electronic supplementary material

Below is the link to the electronic supplementary material.

Supplementary material 1 (DOCX 15 KB)
