# Supplementary material for: Risk prediction of recurrent venous thromboembolism: a multiple genetic risk model
Source: J Thromb Thrombolysis. 2018 Oct 27;47(2):216–26. doi: 10.1007/s11239-018-1762-7 (PMC6394443; doi:10.1007/s11239-018-1762-7)
Supplement: Supplementary file 1 — Supplementary material 1 (DOCX 15 KB) [file 11239_2018_1762_MOESM1_ESM.docx]

**Supplementary Table 1**: Predictive ability of different dichotomized models. Models were calculated by summing the number of risk alleles for different SNPs. The sum is dichotomized into 'low' and 'high' risk groups (Multivariate analyses adjusted for sex, age and family history of VTE)

| **Model** | **Low risk group (n, %)** | **High risk group (n, %)** | **HR (95% CI)** | **Pre-test probability (%)** | **Post-test probability (%) low risk group** | **Post-test probability (%) high risk group** |
| --- | --- | --- | --- | --- | --- | --- |
| **Whole population** |  |  |  |  |  |  |
| 12-SNPs GRS | 0-6 (n = 497, 47.3%) | 7-13 (n = 554, 52.7%) | 2.02 (1.38-2.96) | 12.0 | 8.1 (5.7-10.6) | 15.3 (12.3-18.3) |
| 8-SNPs GRS | 0-4 (n = 507, 48.2%) | 5-11 (n = 544, 51.8%) | 1.88 (1.29-2.75) | 12.0 | 8.4 (6.0-10.9) | 15.2 (12.1-18.2) |
| 5-SNPs GRS | 0-2 (n = 456, 43.4%) | 3-7 (n = 595, 56.6%) | 1.62 (1.11-2.38) | 12.0 | 9.2 (6.5-11.9) | 14.0 (11.2-16.8) |

**12-SNP GRS**: [*FII* (rs1799963), *FV* (rs6025), *ABO* (rs8176719), *ApoM* (rs805297), *F11* (rs2036914), *FGG* (rs2066865), *MRPL37* (rs10888838), *THBD* (rs1042580), *A2M* (rs9939609), *FTO* (rs9939609*), PAI-1 (rs1799889), TFAM (rs1937)].*]. **8-SNP GRS**: [*FII* (rs1799963), *FV* (rs6025), *ABO* (rs8176719), *ApoM* (rs805297), *F11* (rs2036914), *FGG* (rs2066865), *PAI-1(rs1799889)*, *TFAM (rs1937)*].

**5-SNP GRS**, previously described by Van Hylckama et al., 2014.: [*FII* (rs1799963), *FV* (rs6025), *ABO* (rs8176719), *F11* (rs2036914), *FGG* (rs2066865)].

HR = Hazard ratio of recurrent VTE, Pre-test probability = Risk of recurrent VTE before SNPs model. Post-test probability = Risk of recurrent VTE after SNPs model, n= number of risk alleles.

**Supplementary Table 2:** Predictive ability of different categorized models. Models are calculated by summing number of alleles for different SNPs. The sum is dichotomized into 'low', 'medium' and 'high' risk groups.

| **Model** | **Low risk group (n, %)** | **Medium** **risk group (n, %)** | **High** **risk group (n, %)** | **HR medium vs low (95% CI)** | **HR high vs low (95% CI)** | **Pre-test probability (%)** | **Post-test probability (%) low risk group** | **Post-test probability (%) medium risk group** | **Post-test probability (%) high risk group** |
| --- | --- | --- | --- | --- | --- | --- | --- | --- | --- |
| **Whole population** |  |  |  |  |  |  |  |  |  |
| 12-SNPs GRS | 0-5 (n = 304, 28.9%) | 6-9 (n = 654, 62.2%) | 10-13 (n = 93, 8.8%) | 2.01 (1.23-3.27) | 3.91 (2.14-7.17) | 12.0 | 6.6 (3.8-9.4) | 12.8 (10.3-15.4) | 23.7 (15.0-32.3) |
| 8-SNPs GRS | 0-3 (n = 287, 27.3%) | 4-7 (n = 718, 68.3%) | 8-11 (n = 46, 4.4%) | 2.15 (1.30-3.57) | 5.37 (2.63-10.96) | 12.0 | 6.3 (3.5-9.1) | 13.2 (10.8-15.7) | 28.3 (15.2-41.3) |
| 5-SNPs GRS | 0-1 (n = 175, 16.7%) | 2-4 (n = 776, 73.8%) | 5-7 (n = 100, 9.5%) | 1.88 (1.00-3.52) | 4.45 (2.20-9.01) | 12.0 | 6.3 (2.7-9.9) | 11.5 (9.2-13.7) | 26.0 (17.4-34.6) |

**12-SNP GRS**: [*FII* (rs1799963), *FV* (rs6025), *ABO* (rs8176719), *ApoM* (rs805297), *F11* (rs2036914), *FGG* (rs2066865), *MRPL37* (rs10888838), *THBD* (rs1042580), *A2M* (rs9939609), *FTO* (rs9939609*), PAI-1 (rs1799889), TFAM (rs1937)].*]. **8-SNP GRS**: [*FII* (rs1799963), *FV* (rs6025), *ABO* (rs8176719), *ApoM* (rs805297), *F11* (rs2036914), *FGG* (rs2066865), *PAI-1(rs1799889)*, *TFAM (rs1937)*].

**5-SNP GRS**, previously described by Van Hylckama et al., 2014.: [*FII* (rs1799963), *FV* (rs6025), *ABO* (rs8176719), *F11* (rs2036914), *FGG* (rs2066865)].

HR = Hazard ratio of recurrent VTE, Pre-test probability = Risk of recurrent VTE before SNPs model. Post-test probability = Risk of recurrent VTE after SNPs model, n= number of risk alleles.
